# RNAi Screening in *Drosophila* Cells Identifies New Modifiers of Mutant Huntingtin Aggregation

**DOI:** 10.1371/journal.pone.0007275

**Published:** 2009-09-30

**Authors:** Joanna Doumanis, Koji Wada, Yoshihiro Kino, Adrian W. Moore, Nobuyuki Nukina

**Affiliations:** 1 Lab for Structural Neuropathology, RIKEN Brain Science Institute, Wako, Saitama, Japan; 2 Moore Research Unit, RIKEN Brain Science Institute, Wako, Saitama, Japan; Brigham and Women's Hospital/Harvard Medical School, United States of America

## Abstract

The fruitfly *Drosophila melanogaster* is well established as a model system in the study of human neurodegenerative diseases. Utilizing RNAi, we have carried out a high-throughput screen for modifiers of aggregate formation in *Drosophila* larval CNS-derived cells expressing mutant human Huntingtin exon 1 fused to EGFP with an expanded polyglutamine repeat (62Q). 7200 genes, encompassing around 50% of the *Drosophila* genome, were screened, resulting in the identification of 404 candidates that either suppress or enhance aggregation. These candidates were subjected to secondary screening in normal length (18Q)-expressing cells and pruned to remove dsRNAs with greater than 10 off-target effects (OTEs). *De novo* RNAi probes were designed and synthesized for the remaining 68 candidates. Following a tertiary round of screening, 21 high confidence candidates were analyzed *in vivo* for their ability to modify mutant Huntingtin-induced eye degeneration and brain aggregation. We have established useful models for the study of human HD using the fly, and through our RNAi screen, we have identified new modifiers of mutant human Huntingtin aggregation and aggregate formation in the brain. Newly identified modifiers including genes related to nuclear transport, nucleotide processes, and signaling, may be involved in polyglutamine aggregate formation and Huntington disease cascades.

## Introduction

Huntington Disease (HD) is a late-onset, autosomal dominant neurodegenerative disorder characterized at the genetic level by expansion of a CAG repeat in the huntingtin (htt) gene. HD, together with 8 other diseases including the Spinocerebellar Ataxias (SCAs), DRPLA and SBMA, is classified as a CAG repeat disease. Expansion of the CAG repeat in exon 1 of the htt gene to greater than 35 repeats results in a disease-causing expanded polyglutamine tract in the Htt protein. Mutant Htt is aggregation-prone, and acquires a toxic gain-of-function (GOF) by interfering with, or disrupting, normal cellular pathways such as the ubiquitin-proteasome system (UPS), transcriptional regulation, and signaling pathways [1]. In HD, medium spiny neurons of the striatum are selectively affected, leading to the clinical features of the disease including chorea, cognitive abnormalities and psychiatric disturbance.

Researchers have long been aware of the benefits of using *Drosophila melanogaster* to study human disease, particular diseases of the nervous system. The fly is particularly amenable to sophisticated genetic approaches and the ability to temporally and spatially control the expression of transgenes is a powerful advantage of this system. CAG repeat diseases, caused by a toxic GOF, can be modeled in the fly by expression of the mutant protein. *Drosophila* models for SCA 1, SCA 3, MJD, SMBA and DRPLA have been successfully established and mimic various aspects of this group of diseases such as the presence of mutant protein aggregates, progressive neurodegeneration and behavioural abnormalities [2], [3], [4], [5].

In recent years, RNAi has become a powerful approach to study gene loss-of-function (LOF). The relative ease with which genes can be ablated in insect cells has lead researchers to carry out large-scale and even genome-wide analyses to study the effects of gene LOF on various cellular pathways [6], [7]. The problem of off-target-effects (OTEs) has raised some concern over previously published results, and highlighted the importance of thorough validation of candidates identified in large scale RNAi screens through a variety of techniques [8], [9].

We have combined a high throughput RNAi screen with *in vivo* candidate validation to identify potential new regulators of mutant human Htt aggregation. Although there are some controversies about polyglutamine aggregate and cellular toxicity, aggregate formation is a main pathological feature and detecting modulators of aggregation may help our understanding of the pathological process of Huntington disease. By establishing an *in vitro* model of HD in *Drosophila* cells stably expressing htt exon 1 fused to EGFP with an expanded polyQ tract (62Q), we screened 7200 dsRNA molecules for their effects on mutant htt aggregation. We identified 404 candidate dsRNAs that could either suppress or enhance aggregation. We carried out secondary and tertiary screening in our cell culture model to select the strongest candidates and investigated these candidates further using *in vivo* models of HD established for this purpose. Transgenic flies were established that express htt exon1 fused to EGFP with either normal length (18Q) or expanded (62Q or 152Q) and either with or without an NLS for nuclear targeting.

To our knowledge, this is the first *Drosophila* RNAi screen for modifiers of a mutant human aggregating protein. We have identified and thoroughly validated 21 candidate genes in our study using *in vivo* fly models of HD. These candidate genes may shed light on the underlying pathways important in the development and pathogenesis of human HD.

## Results

### Primary high-throughput RNAi screening in cultured cells

**Figure 1:**
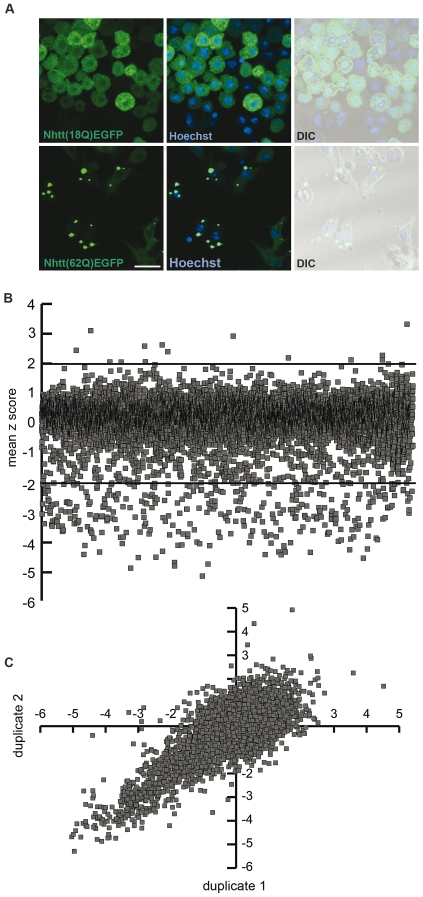
High-throughput RNAi screening in a *Drosophila* cell culture model of HD. *Drosophila* BG2 cells stably expressing Nhtt(18Q)EGFP (top panel) or Nhtt(62Q)EGFP. Induction with CuSO_4_ shows predominantly cytoplasmic localization of Nhtt(18Q)EGFP with some perinuclear accumulation and cytoplasmic puncta. Large, cytoplasmic inclusions form upon expression of Nhtt(62Q)EGFP. Scale bar represents 18.75 µm (A). BG2-Nhtt(62Q)EGFP cells were treated with 7200 dsRNAs and the mean number of inclusions/cell detected by ArrayScan® across duplicate treatments were used to calculate the z score for each dsRNA. The mean z score for each dsRNA is represented as a scatter plot (B). Screen reproducibility across two independent treatments is shown (C).

To utilise high throughput RNAi screening in *Drosophila* cells to identify genes that modify aggregation of polyglutamine-expanded human huntingtin exon 1, we established an *in vitro* cell culture model of human HD as described in Materials and Methods. BG2-c2 cells [24] were chosen to enrich for candidates expressed in neuronal cells. Nhtt(18Q)EGFP predominantly localized to the cytosol with perinuclear accumulation and some punctate staining perhaps indicative of nuclear and other intracellular membrane association [10]. In contrast, Nhtt(62Q)EGFP with a pathogenic number of glutamine residues formed cytoplasmic inclusions within 16 hours of induction (Figure 1A).

For large-scale screening, we needed a rapid method to detect the number of intracellular inclusions in the cell. The Cellomics ArrayScan® is able to detect intracellular objects based on a user-defined protocol. We devised a protocol to count the number of EGFP-positive inclusions and also estimate inclusion size based on the number of pixels of EGFP fluorescence for the objects detected in stable BG2- Nhtt(62Q)EGFP cells. We tested the efficacy of RNAi by treating BG2- Nhtt(18Q)EGFP cells with dsRNA against EGFP. Efficient knockdown was achieved within 48 hours of treatment using the bathing method (Figure S1A) and led to the reduction of both EGFP-positive cells (Figure S1B) and EGFP-positive inclusions (Figure S1C) as detected by ArrayScan® in BG2-Nhtt(62Q)EGFP cells. Given our aim to identify candidate molecules that could modify aggregation of a human disease protein using a *Drosophila* cell culture model, we chose to screen the Open Biosystems RNAi library because it is enriched for *Drosophila* orthologues of mammalian proteins. We prepared dsRNA using dsDNA templates provided in the library, as described in Materials and Methods. Prior to beginning large-scale screening, we ran a test plate through the treatment protocol to ensure that the dsRNAs made were able to ablate gene expression. We selected the library plate containing dsRNA against diap1, a gene essential for the survival of *Drosophila* cells. Cells treated with diap1 dsRNA had significantly reduced cell viability (22% compared with plate average) demonstrating that the method of treatment and quality of dsRNAs was sufficient for screening (Figure S1D). We therefore proceeded to screen the 7200 library dsRNAs (75 96-well plates) in duplicate to identify dsRNAs that could either enhance or suppress Nhtt(62Q)EGFP aggregation using the Cellomics ArrayScan®. Candidates were selected based on their calculated z score, indicating the extent of difference from the plate mean in terms of standard deviations. An outline of our screening approach is shown in Figure S1E.

**Figure 2:**
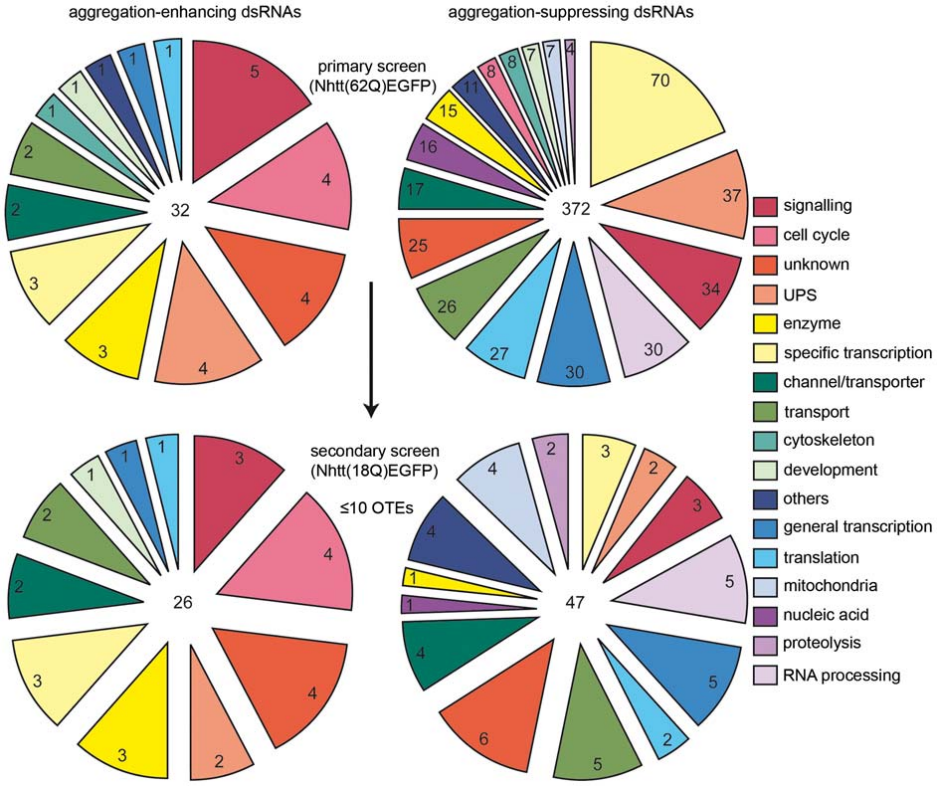
Functional categorization of primary screen candidates. 404 candidates identified through primary RNAi screening were placed into functional groups based on Gene Ontology terms for Biological Process and Molecular Function retrieved from FlyBase and FLIGHT databases. Aggregation-enhancing dsRNAs (left charts) and aggregation-suppressing dsRNAs (right charts) are represented by largely distinct functional groups (top charts). Functional group representations following secondary screening in Nhtt(18Q)EGFP cells, where candidates were pruned to eliminate dsRNAs which non-specifically reduced the expression of Nhtt(18Q)EGFP and those with greater than 10 predicted 19 nt OTEs and shown in the lower charts. Numbers at the centre of each pie chart indicate the total number of candidates and peripheral numbers indicate the number of candidates represented by that category. OTE pruning and elimination of candidates following secondary screening largely reduced the number of aggregate-suppressing dsRNAs, particularly those represented by the specific transcription functional group.

Of the 7200 dsRNAs screened, we identified 404 candidates that modified Nhtt(62Q)EGFP aggregation. Of these, 32 had positive z scores (≥2) indicating that the dsRNA could enhance aggregation, and 372 candidates had negative z scores (≤−2) resulting from suppression of aggregation by dsRNA treatment (Figure 1B). Figure 1C shows the screen reproducibility over the two individual treatment assays. Using FlyBase and FLIGHT database batch download options, we automatically retrieved Gene Ontology (GO) terms for each candidate and then manually assigned functional groups based on this information. Interestingly, functional groups represented by aggregate-suppressing dsRNAs were largely distinct from functional groups of aggregate-enhancing dsRNAs (Figure 2). Our full candidate list with z scores, viability scores and functional group categorization is presented as an excel spreadsheet in Data S1.

### Multiple predicted OTEs contribute to false positives and data skew

Our data showed a strong skew towards candidates with negative z scores; those dsRNAs that suppressed mutant Htt aggregation. Using the FLIGHT database to retrieve the RNAi probe sequence for each of our candidates, and then determining the number of predicted OTEs using the DRSC website tool, we were able to correlate our results with the number of OTEs in our candidate list. Transcription factors (TFs) are notoriously sensitive to potential OTEs due the prevalence of trinucleotide repeats in many TF-encoding genes. Not surprisingly, therefore, we found that the category representing ‘Specific Transcription’ was the most severely reduced when candidates with more than 10 predicted OTEs were excluded (Figure S2). Exclusion of such candidates impacted the aggregate-suppressing candidates most notably, accounting for, in part, the skew in our data. Of note, we found a significant negative correlation between cell viability and the number of predicted OTEs (Figure S3A) adding further caution to growing data concerning the problems of gene off-targeting in *Drosophila* RNAi experiments. Furthermore, dsRNAs with many predicted OTEs are overrepresented amongst our list of candidates, with 44% having more than 10 potential OTEs compared with 5.7% of the entire RNAi library (Figure S3B). It is therefore clear that OTEs contribute to false positive candidates in this library.

### Secondary screening in 18Q cells

To eliminate aggregate-suppressing dsRNAs that reduced the number of aggregates simply by reducing transgene expression in our stable cell line, we treated BG2-Nhtt(18Q)EGFP with dsRNAs identified as candidates in our primary screen. Excepting the different cell line used, treatment conditions were the same as for primary screening and ArrayScan® data for the percentage of EGFP-positive cells and EGFP intensity was obtained. We decided to take a stringent approach to candidate pruning and eliminated candidates that did not reduce the percentage of EGFP-positive cells by more than 10% compared to the average for a LacZ control. Treatment with a LacZ dsRNA alone reduced the % EGFP-positivity compared to untreated cells. At the time of secondary screening, the problem of off-target effects was not fully known. Since this issue came to light, we discovered that the LacZ control dsRNA used in our secondary screen had one predicted off-target. This potentially resulted in the retention of more candidates following secondary screening because the LacZ control showed some decrease in EGFP positivity. We therefore designed a new LacZ control dsRNA (NewLacZ) for all subsequent tertiary screening and biochemical analyses *in vitro*. We also confirmed that the potential off-target gene (pcx) had some effect on EGFP expression and aggregation (data not shown). We could not distinguish between candidate dsRNAs that reduced the Nhtt(18Q)EGFP fluorescence by enhancing proteolytic pathways or by general transcriptional reduction. Thus, some genuine candidates may have been excluded following secondary screening. Functional groups of enhancer and suppressor dsRNA following OTE pruning and secondary screening are shown in Figure 2 (lower charts).

### Tertiary screening using *de novo* designed RNAi probes

To further confirm the effect of candidate dsRNAs that passed through previous rounds of screening, we independently designed and synthesized dsRNA probes, spanning a different region of the gene from the library dsRNA. *De novo* probes were designed using the E-RNAi software [11]. Primer pairs were selected for their specificity, and for their ability to target all isoforms of a given gene. The size and integrity of PCR amplicons and synthesized dsRNAs were checked by non-denaturing and denaturing agarose gel electrophoresis respectively (Figure S4A). Details of primer design and synthesis of dsRNA probes can be found in Supporting Methods. In the primary screen, we used a relatively high concentration of dsRNA (100 nM). We treated cells with two concentrations, one estimated to be 108 nM, similar to primary screen treatment concentration, and the other lower concentration 43 nM, slightly above the optimal concentration reported [7]. We initially carried out RT-PCR using Htt primers to confirm that dsRNAs do not affect Htt expression and did not see a noticeable difference in expression level of the Htt transgene (data not shown).

**Figure 3:**
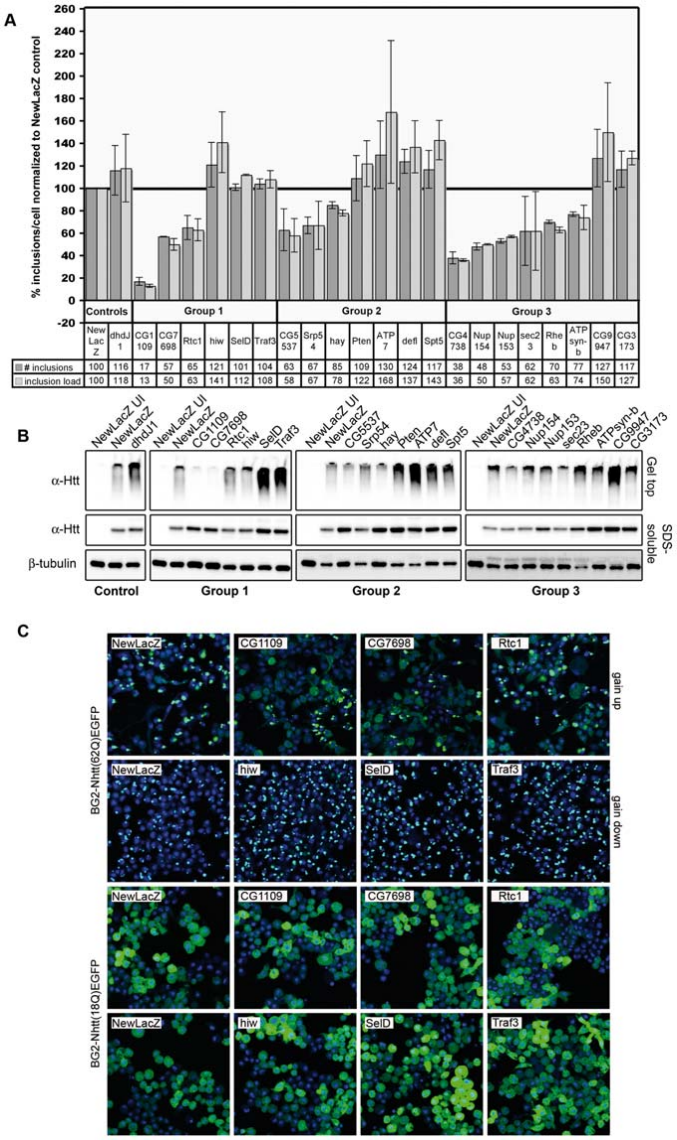
ArrayScan® data, Western Blotting and confocal analysis of selected candidates. Following secondary screening and OTE pruning, *de novo* dsRNA probes were designed for the remaining candidates. BG2-Nhtt(62Q)EGFP cells and BG2-Nhtt(18Q)EGFP cells were treated in duplicate with these *de novo* probes. Candidate groups 1 to 3 ArrayScan® data for the mean number of inclusions/cell and the mean inclusion load/cell (# inclusions X mean inclusion size/# cells) as a percentage of the NewLacZ control dsRNA are shown. dsRNA against dhdJ1 was used as a control. Error bars represent +/− SD. (A). BG2-Nhtt(62Q)EGFP cells treated with *de novo* dsRNAs were analyzed biochemically by Western blotting with α-Htt antibody (EM48). SDS-insoluble (gel-top) material is shown in the top panel (B). BG2-Nhtt(62Q)EGFP cells and BG2-Nhtt(18Q)EGFP treated with *de novo* dsRNAs were analyzed by confocal microscopy. Confocal images for group 1 candidates are shown (C). For imaging aggregation-suppressing dsRNAs in BG2-Nhtt(62Q)EGFP cells, EGFP gain was increased in order to show the increase in cells with diffuse expression compared to the NewLacZ control. EGFP gain was reduced for imaging aggregate-enhancing dsRNAs due to the intensity of fluorescence in highly-aggregating cells.

Tertiary screen candidates were also tested for their effects on the expression level of BG2-Nhtt(18Q)EGFP cells. Based on tertiary screening results, we selected 21 candidates that modified mutant Htt aggregation in the direction consistent with primary screening results and eliminated candidates that drastically altered EGFP fluorescence. These 21 candidates were placed into 3 groups, based on the percentage difference in expression level of normal length htt (18Q). Group 1 candidates differed in 18Q expression by no more than 10%, group 2 by no more than 20% and group 3 by no more than 30% (Table 1). ArrayScan® results for candidate groups 1–3 are shown in Figure 3A and the level of target gene knockdown by dsRNA is demonstrated by RTPCR in Figure S4B. Confocal images of BG2-Nhtt(62Q)EGFP and Nhtt(18Q)EGFP cells treated with group 1 candidate modifiers are shown in Figure 3C. Figure S5A shows confocal images for the 12 aggregate-suppressing dsRNAs and Figure S5B shows the 9 aggregate-enhancing dsRNAs.

**Table 1:** Candidate genes that modify mutant Nhtt(62Q)EGFP aggregation.

Group	FB Number	Gene Sym	Gene Name	Functional category	Fold change (# inclusions)	Fold change (inclusion load)	% viability	Human homologue	Mouse homologue
1	FBgn0046222	CG1109	CG1109	Unknown	0.17	0.6	95	WDR33	Wdr33
1	FBgn0038636	CG7698	CG7698	RNA processing	0.57	0.5	114	CPSF3	Cpsf3
1	FBgn0020909	Rtc1	Rtc1	RNA processing	0.65	0.63	87	RCL1	Rcl1
1	FBgn0030600	hiw	highwire	UPS	1.21	1.41	117.5	MYCBP2	Phr1
1	FBgn0020615	SelD	Selenide, water dikinase	Enzyme	1.08	1.13	97	ENSG00000182722	Sephys1
1	FBgn0030748	Traf3	TNR-receptor-associated factor 3	Signaling	1.04	1.08	99.5	TRAF3	Traf3
2	FBgn0035639	CG5537	CG5537	Nucleic acid	0.63	0.58	90	UPRT	Uprt
2	FBgn0024285	Srp54	Srp54	RNA processing	0.67	0.67	62	SFRS12	Sfrs12
2	FBgn0001179	hay	haywire	General transcription	0.85	0.78	97	ERCC3	Ercc3
2	FBgn0026379	Pten	Pten	Signaling	1.09	1.22	103	PTEN	Pten
2	FBgn0030343	ATP7	ATP7	channel/transporter	1.3	1.68	106	ATP7a	Atp7a
2	FBgn0036038	defl	deflated	Unknown	1.24	1.37	98.5	INTS7	Ints7
2	FBgn0040273	Spt5	Spt5	General transcription	1.17	1.43	80.5	SUPT5H	Supt5h
3	FBgn0032347	CG4738	CG4738	Transport	0.38	0.36	60	NUP160	Nup160
3	FBgn0021761	Nup154	Nup154	Transport	0.48	0.5	74	NUP155	Nup155
3	FBgn0061200	Nup153	Nup153	Transport	0.53	0.57	73.5	NUP153	Nup153
3	FBgn0037357	sec23	Sec23	Transport	0.62	0.62	98	SEC23A	Sec23a
3	FBgn0041191	Rheb	Rheb	others (autophagy)	0.7	0.63	105	RHEB	Rheb
3	FBgn0019644	ATPsyn-b	ATP synthase, subunit b	Mitochondria	0.77	0.74	81.5	ATP5F1 and MAF	Atp5f1
3	FBgn0030752	CG9947	CG9947	Cell cycle	1.27	1.5	101	TMEM30A	Tmem30A
3	FBgn0034964	CG3173	CG3173	Translation	1.17	1.27	85.5	ENSG00000164880	Inst1

Following primary and secondary screening in Nhtt(62Q)EGFP and Nhtt(18Q)EGFP cells respectively, pruning for OTEs, and tertiary screening with *de novo* dsRNA probes in both cell lines, final candidates were placed into three groups. All candidates consistently altered mutant Htt aggregation in cell culture screening rounds. Group 1 candidates did not change the EGFP intensity of Nhtt(18Q)EGFP in the tertiary screen by more than 10%, group 2 by no more than 20% and group 3 by no more than 30%. The tertiary screen results for each candidate group are shown as the fold change in the number of inclusions (# inclusions), inclusion load and viability compared to a non-specific dsRNA control. Human and mouse homologues for each *Drosophila* candidate gene are listed.

### Biochemical validation of aggregation

To further validate the effect of each candidate on mutant Htt aggregation, we treated BG2-Nhtt(62Q)EGFP cells with candidate groups 1–3 dsRNAs and prepared whole cell lysates for Western blotting. Although the immunoreactive signal of SDS-insoluble Nhtt(62Q)EGFP residing the gel top is only a qualitative way to assess the amount of mutant Htt aggregation, we consistently observed differences in the level of gel top material by treatment with our candidate dsRNAs (Figure 3B), adding further confidence to results obtained by ArrayScan®.

### Validation of RNAi screen candidates in the *Drosophila* compound eye

**Figure 4:**
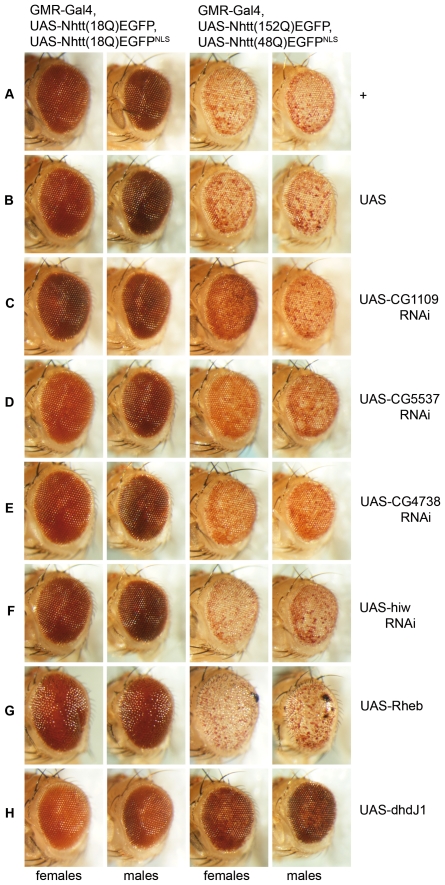
Selected screen candidates modify mutant Htt-induced toxicity in the fly eye. GMR-Gal4-driven expression of UAS-Nhtt(152Q)EGFP, UAS-Nhtt(48Q)^NLS^ in the compound eye results in a progressive loss of pigmentation compared to expression of UAS-Nhtt(18Q)EGFP, UAS-Nhtt(18Q)^NLS^ (A). Carrying an extra UAS transgene does not noticeably alter this phenotype (B). Female flies carrying a UAS-CG1109 RNAi transgene on the X chromosome show a clear suppression of the mutant Htt-induced toxicity phenotype, whereas no clear modification is seen in male flies lacking the UAS-CG1109 RNAi transgene (C). Expression of UAS-CG5537 RNAi on the X chromsome (D), and an autosomal UAS-CG4738 RNAi transgene (E) also suppress the mutant Htt-induced degenerative phenotype, with a rescue of pigmentation in these flies. Note: both males and females carry a copy of the for the UAS-CG5537 RNAi transgene as explained in Materials and Methods. Flies carrying a UAS-hiw RNAi transgene showed a slight rough eye phenotype that was also observed when coexpressed with normal Htt (F). Overexpression of Rheb resulted in a drastic enhancement of the Htt phenotype with an increase in black necrotic patches, further loss of pigmentation and a rough eye with bristle disorganization (G). Overexpression of Rheb in flies expressing normal Htt also resulted in a rough eye phenotype with a mild loss of pigmentation. The chaperone molecule, dhdJ1 clearly suppressed Htt-induced loss of pigmentation when overexpressed (H). All flies were aged between 21 and 22 days.

In order to follow up interesting candidates *in vivo*, we established transgenic flies for expression of EGFP-tagged Nhtt with either a normal (18Q) or expanded (152Q) polyglutamine tract and either lacking or containing a nuclear localization signal (NLS). We established UAS-NhttEGFP lines for driving transgene expression in various cell types. We have found that heterozygous expression of non-nuclear mutant Htt using the GMR-Gal4 driver does not produce a strong external eye phenotype, compared to a moderate loss of pigmentation phenotype caused by NLS-containing mutant Htt (data not shown). For genetic modification of Htt-induced cellular toxicity, we established a *Drosophila* line that expresses mutant Htt in both the cytoplasm and nucleus of all neurons and other cell types of the developing and adult compound eye using the GMR-Gal4 driver. We confirmed the presence of Nhtt(48Q)EGFP^NLS^ inclusions in the nucleus and Nhtt(152Q)EGFP inclusions in the cytosol by confocal microscopic imaging of 3^rd^ instar larval eye discs (Figure S6). These flies produce a degenerative eye phenotype characterized by loss of pigment cells and an increased occurrence of dark necrotic spots on the external eye while flies expressing Nhtt(18Q)EGFP/Nhtt(18Q)EGFP^NLS^ have a normal eye phenotype at the same age (Figure 4A). There was no noticeable alteration of this phenotype in flies carrying an extra UAS insertion (Figure 4B).

We took advantage of the availability of UAS-dsRNA lines provided by the VDRC stock centre in Vienna, Austria [12] and the NIG stock centre in Mishima, Japan. Where RNAi stocks were not available, overexpression lines were obtained from the Bloomington stock centre. Our flies, expressing mutant Htt in the eye, were crossed to candidate RNAi or overexpression (OE) stocks. We observed a significant degree of variation amongst the 3 week old progeny in some crosses (data not shown for all candidates). Therefore, we selected the strongest, most consistent candidates that enhanced or suppressed the eye phenotype caused by mutant Htt to test again using the same model.

The novel gene, CG1109, most strongly suppressed Nhtt(62Q)EGFP aggregation in cultured cells when ablated by RNAi (Figure 3). CG1109 RNAi also strongly suppressed mutant Htt-induced toxicity *in vivo* in the fly eye (Figure 4C). The UAS-CG1109 RNAi transgene is carried on the X chromosome. Thus, using males carrying this transgene crossed to our HD females, progeny females carried the UAS-CG1109 RNAi transgene and demonstrated a strong suppression of the mutant Htt-induced phenotype compared to males that lacked the UAS-CG1109 RNAi transgene (Figure 4C). We found that dsRNAs targeting CG5537 and CG4738, the *Drosophila* homologue of Nup160, could suppress, to some extent, the loss of pigmentation phenotype in these flies (Figure 4D and 4E respectively). Furthermore, we observed a clear decrease in the number of necrotic fly eyes in these progeny (data not shown). Although UAS-Hiw RNAi showed some enhancement of the eye phenotype in the preliminary round of screening, we were unable to obtain consistent results with this candidate. However, a slight rough eye with disruption to eye bristles was observed in a mutant Htt-independent manner (Figure 4F). Overexpression of Rheb enhanced the phenotype of mutant Htt flies, with a severe rough eye phenotype and enhancement of pigment cell loss (Figure 4G right panels). However, overexpression of Rheb in flies expressing our NhttEGFP transgene with a normal length polyQ repeat (Figure 4G left panels) or expression of UAS-Rheb alone (data not shown) also resulted in a rough eye phenotype with bristle disorganization. As a control, we used the overexpression of the *Drosophila* homologue of heat shock protein 40, dhdJ1, to suppress the mutant Htt eye phenotype (Figure 4H).

### Validation of RNAi screen candidates in aged adult fly brain

**Figure 5:**
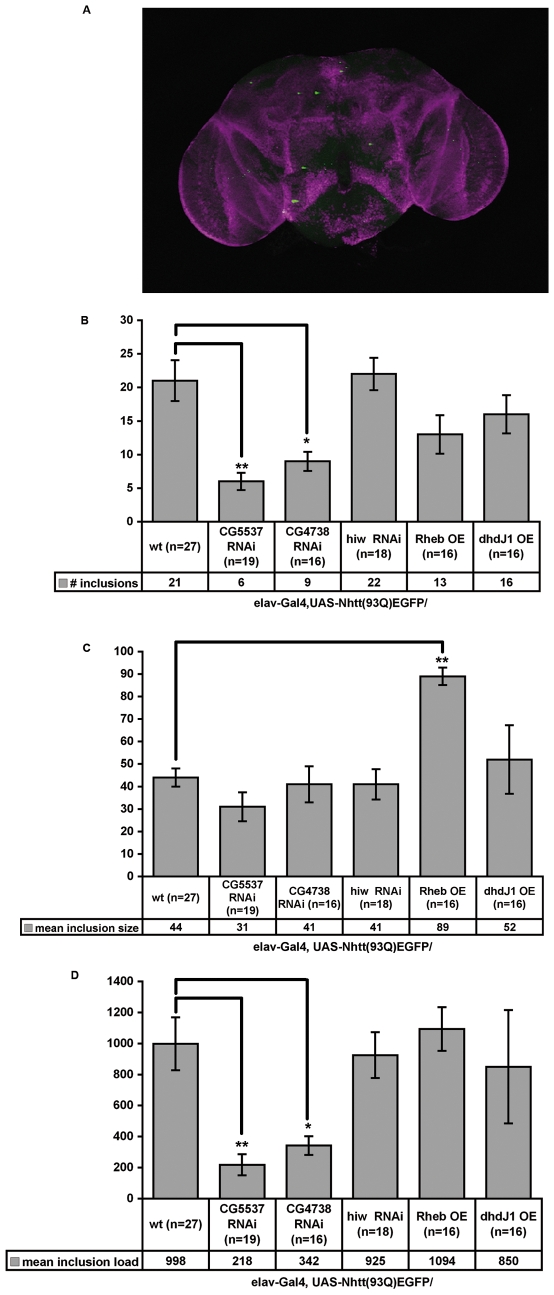
CG5537 and CG4738 RNAi transgenese suppress Htt(93Q)EGFP aggregation in aged *Drosophila* brain. 4-week old (28–29 days) fly brains were dissected from male flies and stained with α-elav antibody (magenta), imaged by confocal microscopy and 5 µm z stacks projected into one image (A). Compared to elav-Gal4, UAS-Nhtt(93Q)EGFP/wt flies, flies carrying a UAS-CG5537 RNAi or UAS-CG4738 RNAi transgene show a significant reduction in the mean number of inclusions (B) and the mean overall inclusion load (# inclusions X mean inclusion size) (D) with no significant reduction in inclusion size observed (C). UAS-hiw RNAi and UAS-dhdJ1 had no significant effect on the number, size or load of inclusions (B–D respectively). UAS-Rheb significantly increased the mean inclusion size (C), however there was an insignificant reduction in inclusion number (A) with no significant change in overall inclusion load (D). Error bars represent +/− SEM. The total number of brains imaged and scored from 1 to 3 experiments are indicated in the data box. ** p≤0.0008, * p≤0.008, Student's t test.

We further confirmed the validity of CG5537 and CG4738 RNAi by quantification of mutant Nhtt(98Q)EGFP brain aggregates. 4 week old male brains, from Elav-Gal4, UAS- Nhtt(98Q)EGFP recombinant lines were dissected and stained with anti-elav antibody and Hoechst 33345. We quantified the number and size of brain aggregates from stacked images of 5 µm confocal sections, using ImageJ software. We observed a significant decrease in the average number of visible inclusions in each brain by expression of UAS-CG4738 RNAi and UAS-CG5537 RNAi (Figure 5B and 5C). Although Rheb overexpression did not significantly increase the number of brain inclusions or the inclusion load, the average aggregate size was increased (Figure 5C).

In summary, by using various *in vivo* models of HD we have validated the effect of several candidate genes identified through a large-scale RNAi screen for modifiers of mutant Htt aggregation.

## Discussion

To make use of the amenability of cultured *Drosophila* cells to gene knockdown by RNAi, we aimed to screen a library of dsRNA molecules covering around half of the fly genome and enriched for fly genes with mammalian homologues, for modifiers of Htt(62Q)EGFP aggregation. We carried out several rounds of screening to identify such modifier dsRNAs and carefully confirmed our screen results by addressing issues such as non-specific transcript reduction and potential gene off-targeting. Following our stringent screening method, our final 3 groups of candidates were assessed biochemically by Western Blot analysis and visually by confocal microscopy.

Our candidates fell into several functional groups, the most notable being transport molecules, including those involved in nuclear transport, and nucleotide processing including RNA metabolism. Nuclear transport has been reported to be important in mutant Htt pathogenesis, with Htt itself reportedly shuttling between the nucleus and the cytoplasm [10], [13]. RNA metabolic processes are receiving increasing attention in the neurodegenerative field and RNA binding proteins have been identified as polyglutamine aggregate-interacting proteins and contributing to polyglutamine disease pathogenesis [14], [15]. Although we identified many genes through RNAi screening with predicted involvement in the Ubiquitin Proteasome System (UPS), most were aggregation-suppressing. This may be due to the essential role of many of these genes, most of which were eliminated by OTE pruning and secondary screening for reduction of Nhtt(18Q)EGFP, although we cannot exclude the possibility that some eliminated dsRNAs might reduce Nhtt(18Q)EGFP expression by enhancing cellular proteolytic activities. Loss of the ubiquitin ligase highwire consistently resulted in an increase in mutant Htt inclusion number and size in our cell culture model of HD and appeared to enhance the eye degeneration phenotype of mutant Htt, although the high degree of phenotypic variability with the UAS-hiw RNAi lines used made it difficult to reach a sure conclusion on the role of this gene in HD pathogenesis. Furthermore, no significant difference was found in the number or size of inclusions in aged adult brains expressing UAS-hiw RNAi (Figure 5).

Our screen was biased towards detecting suppressors due to the high aggregation propensity of our cell line. Investigation of aggregation-enhancing dsRNAs with z scores slightly below our +2SD cut-off (z scores between 1.5 and 2) revealed several UPS dsRNAs of the ubiquitin-ligase class. This is consistent with the role played by the UPS system in targeting mutant Htt for degradation. This group of enhancers can be viewed as an excel spreadsheet in Data S1.

To further validate our high-confidence candidates, we established *in vivo* models of HD to investigate whether or not these genes are likely to be involved in mutant Htt toxicity and/or modification of mutant Htt aggregation *in vivo*. The *Drosophila* compound eye has long been utilized to assess potential genetic interactions particularly in the fields of apoptosis and degeneration in part because the eye is sensitive to cell loss and produces a visible phenotype when the highly organized ommatidial structure is disrupted. Furthermore, the eye is not essential for viability, allowing genetic interactions to be investigated in the adult, even with highly toxic gene products. We were able to validate the role of several of our gene candidates using the fly eye as a model system. Expression of UAS-CG4738 RNAi and UAS-CG5537 RNAi transgenes resulted in a noticeable and consistent suppression of the loss of pigmentation phenotype caused by heterozygous expression of Nhtt(48Q)EGFP^NLS^ and Nhtt(152Q)EGFP (Figure 4). We also observed a strong suppression of this phenotype with UAS-CG1109 RNAi and a strong enhancement of the phenotype by overexpression of Rheb, consistent with the role of autophagy in Htt aggregation [16], [17].

Limiting our *in vivo* validation of candidates to the eye raised several problems. Although the eye is valuable as a toxicity model, the relationship between polyglutamine aggregation and toxicity is not clear in the eye. Overexpression of the heat shock protein 40 homologue, dhdJ1, demonstrates a drastic suppression of ataxin 1-induced toxicity [18] and in our hands, this same transgene resulted in suppression of mutant Htt-induced toxicity. However, suppression of polyglutamine toxicity was independent of a visible change in ataxin-1 aggregation [18]. We therefore set out to assess the role of selected candidates in aggregation of mutant Htt in aged adult fly brain. Our aggregation model, expressing one copy of a UAS-Nhtt(93Q)EGFP transgene using the pan-neuronal driver, elav-Gal4, formed visible inclusions in the brain and optic lobes (Figure 5A). Although all progeny differed in age by no more than 24 hours, there was a high degree of variation in the number and size of visible inclusions. Nevertheless, we found a significant reduction in the number of inclusions and overall inclusion load by expression of UAS-CG4738 RNAi and UAS-CG5537 RNAi. We could not obtain a clear brain aggregation result for CG1109 due to poor brain quality for all progeny from this cross. CG1109 is a novel gene, recently identified in a primary neuronal RNAi screen for genes required for neurogenesis [19]. CG1109 may therefore be essential for neuron function and pan-neuronal expression of an RNAi construct may be toxic.

We were unable to detect a significant reduction in mutant Htt inclusions in the brain by overexpression of dhdJ1. It is possible that dhdJ1 requires some cofactor for its function as a heat shock protein that may be lacking in elav-positive neurons in the fly brain. However, given that so many studies use the eye for validation of polyglutamine modifiers, it should be noted that perhaps not all such modifiers will prove valid in models for mutant Htt aggregation. Our strongest candidates, CG4738 and CG5537 were validated by two independent RNAi stocks in the eye model (Figure 4 and data not shown).

Although some corresponding results were observed between suppressor activity in the cellular system and reducing toxicity in eye, the discrepancy is not unexpected. Even in our cellular system, based on the inclusion formation and cell viability, a distinct correlation was not confirmed (Table 1, Figure S7). Previously, drugs have been screened for their effect on polyglutamine aggregate formation using cellular models. One drug inhibited aggregation and suppressed neurodegeration [20], [21]. Another compound was reported to promote inclusion formation and prevent the huntingtin-mediated proteasome dysfunction, which is related to cell toxicity [22], [23]. These results and the existence of heterogeneous aggregate species such as fibrils and oligomers suggest that the decrease of inclusions might correlate to the change of some specific toxic species of aggregates depending on the system used.

We further examined the functional relationship among mouse homologues of the selected candidates (Figure S8). The main gene group includes nucleotide processing, nucleoporin and signaling. The signaling genes are related to the autophagy system, which could degrade polyglutamine aggregates. The role of other major groups on aggregate formation, such as genes for nucleotide processing and nucleoporin, is unknown. Since the main localization of these gene products is nucleus, their function may be related to the formation of nuclear inclusions. Reduction of the nucleoporin 160 protein (CG4738) consistently rescued Htt-induced toxicity and aggregation in our cell line and *in vivo*. It is feasible, however, that a nucleoporin may act as a docking site for the accumulation and aggregation of mutant Htt. Further work remains to elucidate the role of our candidates in the mammalian system and their mechanism of action.

In summary, we have carried out a thorough screen for modifiers of mutant human Htt aggregation using new models of HD established in cultured *Drosophila* cells and in the fly. Further investigation of our candidates, particularly those involved in nucleotide processesing and intracellular transport, including nuclear transport may uncover, as yet, unexplored pathways relevant to human Huntington Disease pathogenesis.

## Methods

### Cloning of *Drosophila* constructs

We cloned N-terminal Htt exon 1 with 18Q, 62Q or 152Q as fusions with EGFP and containing a C terminal NLS and MYC tag into *Drosophila* expression vectors as described in Supporting Information.

### Establishment of stable, inducible *Drosophila* cells expressing Htt exon 1-EGFP

We established stable, single colony-isolated BG2 cell lines using the DES® system (Invitrogen) as per the manufacturer's instructions. Briefly, larval central nervous system-derived parental cells, BG2-c2 cells [24] were cotransfected with copper-inducible pRMHa3-NhttEGFP encoding either an 18Q or a 62Q repeat together with pCoBlast to confer Blasticidin-resistance. Stably-integrated heterogeneous cells were selected in the presence of 25 ug/ml Blasticidin. 1–3 cells were seeded into 96 well plates and isolated colonies were picked and expanded. Individual clones were checked for expression of the NhttEGFP transgene by microscopy and Western blot analysis following induction with CuSO_4_. Cells were cultured in Schneider's *Drosophila* Medium (Invitrogen) supplemented with 10% heat-inactivated FBS (Sigma) and 10 µg/ml insulin (Sigma). Medium was supplemented with 0.5 mM CuSO_4_ for induction of NhttEGFP expression. Following initial selection, HD cell lines were not maintained in Blasticidin.

### dsRNA library synthesis

*In vitro* transcription reactions were set up in 96 well U-bottom plates (Cellstar) using Ambion T7 megascript kits to simultaneously synthesize sense and antisense RNA strands in one reaction and purified as described in Text S1.

### ArrayScan® analysis

Fixed, stained cells were analyzed by ArrayScan®V^TI^ High Content Screening (HSC) Reader (Cellomics, Pittsburgh, PA, USA) using Target Activation Bio Application (TABa). TABa analyzes images acquired by an HSC Reader and provides measurements of the intracellular fluorescence intensity and localization on a cell-by-cell basis.

In each well, several thousand cells were counted and quantified for the number and size of Nhtt(62)EGFP inclusions. Nuclei stained by Hoechst 33285 provided the autofocus target and scored the number of quantified cells. Screening consisted of two scans using Hoechst and FITC (for EGFP) fluorescence. At first, the number of aggregates was calculated. Fluorescent spots of at least 5 pixels in size (magnification 40×) with an average EGFP intensity of more than 1500 were labeled as inclusions. Secondly, nuclei were defined as the objects of interest and their number was determined. EGFP intensity in each cell was calculated in the perinuclear region within the distance of 3 pixels from the nucleus and when the average intensity exceeded 250, the cell was considered as EGFP-positive. The percentage of the cells with aggregates was calculated.

ArrayScan® data was used to calculate the number of inclusions per cell (inclusion #/cell) and the inclusion load per cell (inclusion load/cell), which takes into account the inclusion size (inclusion number multiplied by inclusion size and divided by the total number of cells).

### Large scale RNAi screening

Detailed methods of our large-scale RNAi screen including methodology can be found in Text S1.

### Fly stocks

NhttEGFP transgenes with either an 18Q or 152Q polyglutamine repeat and either with or without a nuclear localization signal (NLS) were subcloned into pUAST plasmid and injected by standard methods into w^1118^ embryos to establish transgenic flies. Driver lines used in our analysis were obtained from the Bloomington stock centre and were recombined with our transgene(s) for stable expression. Due to CAG repeat instability, some fly stocks were injected with constructs with repeat lengths other than 152Q. In such cases, where expressed protein sizes assessed by Western blotting were inconsistent with a 152Q repeat, the transgene was amplified by RT-PCR and the CAG repeat sequenced. UAS-dhdJ1 have been previously described and were kindly provided by Prof. Kazemi-Esfarjani [18]. RNAi flies were obtained from the VDRC stock centre [12] in Vienna and the NIG stock centre in Mishima. UAS-Rheb flies and the GMR-Gal4 and elav-Gal4 driver lines were obtained from the Bloomington stock centre. For all autosomal insertion lines and UAS-CG1109 RNAi, we set up crosses with virgin females carrying our Htt transgenes to males carrying the candidate transgene. For crosses with UAS-CG5537 RNAi, virgin females were crossed to HD males.

### Prediction of Off-Target Effects

The amplicon sequence for each dsRNA in the Open Biosystems library was automatically retrieved from the FLIGHT database and entered into the *Drosophila* Resource Screening Center (DRSC) OTE search tool to predict the number of potential Off-Target Effects with a 19 nt match.

### *De novo* design of amplicons for RNAi

The transcript sequences for the final candidates were retrieved from FlyBase and the region targeted by the Open Biosystems dsRNA was manually highlighted. Transcript sequence not targeted by the library was used to design T7-tagged oligos for amplication of *de novo* RNAi probes using the E-RNAi tool (Heidlberg, Germany). Where possible, probes were selected to target all possible transcripts of a given gene and had no predicted 21 nt OTEs.

### Synthesis of *de novo* RNAi probes

To prepare the template for PCR amplification of the target regions for *in vitro* transcription (IVT), total RNA was prepared from 200 liquid N_2_ freeze-dried whole w^1118^ flies using TRIzol® reagent (Invitrogen). Oligo d(T)-primed cDNA was synthesized from 2 µg total RNA, using First Strand cDNA synthesis kit according to the Manufacturer's directions (Novagen). 3 µl were used in a standard PCR reaction using KODPlus DNA polymerase (TOYOBO, Japan) and using the primer pairs designed as described above and synthesized by Operon. PCR products were purified using the Vacuum Manifold® system (Millipore) and a sample was checked by agarose gel electroporesis. Purified PCR products containing T7 promoters at each end were used as templates for *in vitro* transcription using the Megascript T7 kit (Ambion) according to the manufacturer's directions. dsRNA is automatically made as each strand is synthesized in a single reaction. dsRNAs were purified using the Millipore Vacuum Manifold system and the concentration calculated by spectrophotometry. In cases where multiple bands were observed in the PCR product, the band of the correct size was excised and purified using the Wizard® Gel and PCR Clean-Up kit (Promega) before being used as a template for IVT.

### Tertiary Screening using *de novo* dsRNAs

Based on calculations of Clemens *et al*, 2000 we brought each dsRNA to 860 nM stock and aliquoted the appropriate amounts for 43 nM and 108 nM treatments into 96 well plates. Cells were treated with each *de novo* dsRNA probe in duplicate on separate experimental days and in different well positions. Each plate was arrayed with controls against NewLacZ, diap1 and dhdJ1 (hsp40). Scores were normalized by dividing the inclusions/cell value by the NewLacZ control value. Averaged, normalized scores for # inclusions/cell and inclusion load/cell were calculated.

### Western Blot analysis

BG2-Nhtt(62Q)EGFP cells were treated with 43 nM dsRNAs for 48 hours in 12 well plates and the culture medium was replaced with induction medium containing 0.5 mM CuSO_4_ for 16 hours. Cells were washed in PBS and then harvested in 1% SDS/PBS supplemented with Complete® Protease Inhibitor Cocktail (Roche), and divided into two for WB analysis and prepareation of total RNA. Cells for WB analysis were lysed by sonication, gently centrifuged and the protein concentration measured by BCA assay. 3 µg of whole cell lysates were boiled in LDS sample buffer/DTT and electrophoresed at 200 V through a 4–12% NuPAGE gel (Invitrogen) in MOPS buffer. Proteins were transferred wet onto PVDF (Millipore) membrane, blocked in 10% skim milk and blotted with EM48 MAB5374 Huntingtin primary antibody (Millipore) and Mouse IgG Peroxidase (GE healthcare) secondary antibody before detection using ECL. Images were captured using LAS-1000 (Fujifilm). Blots were stripped and re-probed with E7 β-tubulin antibody (Hybridoma Bank) and Mouse IgG Peroxidase (GE). The presence of aggregates in whole cell lysates makes quantification difficult, resulting in some apparent loading differences between samples.

### Imaging of adult fly eyes

Fly progeny were collected every 24 hours over a 5 day period and aged for 3 weeks (21–22 days). Flies were randomly selected, anesthetized with CO_2_ and decapitated. Fly heads were aligned on a slide for imaging the left eye and viewed using an Olympus SZX16 dissecting microscope and an external light source (Kenko). Images were captured using a NIKON digital sight DS-L1 camera.

### *In vivo* scoring of aggregation

For quantification of inclusions in the adult brain, brains from male flies aged for 4 weeks (28–29 days) were dissected in PBS and immediately fixed in 4% PFA for 30–60 minutes. Brains were fixed in paraformaldehyde and stained with elav antibody (Developmental Hybridoma Bank, Iowa, USA). Using a 10X objective lens, 5 µm z sections were imaged using a SP2nLeica Confocal and images were converted to greyscale JPEG images using Photoshop and then opened in ImageJ [25] for quantification of aggregation. In some cases, highly fluorescent areas clearly not inclusions, were removed to avoid artificial inclusion counts. The number and size of Htt(93Q)EGFP inclusions were quantified using ImageJ software on greyscale brain images captured by confocal microscopy using the green channel only. Threshold settings were set to a minimum of 100 and the default maximum (255). Using the ‘Analyze Particles’ option, the inclusion number and average inclusion size were calculated. We were unable to confirm expression levels of our candidates in adult brain by RTPCR, probably because contribution of non-RNAi targeted cell types contributing to the total RNA prepared could mask any reduction in elav-positive cells. We were able to detect the overexpression of dhdJ1 and Rheb in the eye model, suggesting that these transgenes are effectively overexpressing these genes (data not shown).

## Supporting Information

Text S1 Supporting Materials and Methods
(0.03 MB DOC)

Figure S1 RNAi screening validation and overview. The efficacy of RNAi treatment in a Drosophila cell culture model of HD was tested using dsRNA against GFP. Cells expressing BG2-Nhtt(18Q)EGFP cells treated with 37 nM GFP dsRNA for 48 hours and visualized by fluorescence microscopy show ablation of GFP (A). BG2-Nhtt(62Q)EGFP cells were treated with GFP dsRNA and analyzed by ArrayScan®. Reduction of Nhtt(62Q)EGFP by GFP dsRNA reduced the number of EGFP-positive cells (B) and the number of EGFP-positive intracellular inclusions (C) detected by ArrayScan®. Screening in 96 well plate format was validated using a screen plate arrayed with random dsRNAs including dsRNA against diap1. Loss of diap1 results in widespread apoptosis as shown by the reduced viability in cells treated with diap1 dsRNA (D). An overview of our approach to screening for modifiers of mutant Htt aggregation, including several rounds of screening in vitro, followed by validation in vivo, is shown (E).
(2.33 MB TIF)

Figure S2 OTE pruning following primary screening. Top pie charts show the functional categorization of all candidates following primary screening in BG2-Nhtt(62)EGFP cells. These candidates were pruned to eliminated dsRNAs with more than 10 predicted OTEs. The Specific Transcription category, including many transcription factors (TFs) was the most drastically reduced following OTE pruning, consistent with the fact that many TFs have repetitive trinucleotide repeats that are sensitive to off-targeting.
(1.81 MB TIF)

Figure S3 Off-target effects reduce cell viability and contribute to false positive candidates. The mean cell viability values for candidates following primary screening were plotted against the number of predicted 19 nt OTEs, demonstrating a significant negative correlation between cell viability and the number of potential OTEs (A). dsRNAs with multiple predicted OTEs are over-represented amongst our candidates following the primary screen. 54% of the dsRNA target sequences in the Open Biosystems library have no predicted 19 nt OTEs, with only 5.7% having more than 10 predicted OTEs (B). In contrast, 30% of our candidates lacked any predicted OTEs, with 44% having greater than 10 potential off-targets (C). The presence of off-target sequences causes inconsistencies in assay results. The percentage of candidates that modified Nhtt(62Q)EGFP consistently from the primary screen, using library dsRNAs, and the tertiary screen, using de novo designed dsRNAs are shown in yellow. The percentage of candidates producing an opposite effect is shown in green, while pink shows the percentage of candidates that gave inconsistent results within the tertiary screen duplicates using de novo dsRNAs with no 21 nt OTEs. The majority of candidates with no more than 1 OTE consistently modified mutant Htt aggregation in vitro. Increasing OTEs increased the likelihood of inconsistent results (D).
(2.19 MB TIF)

Figure S4 de novo RNAi probes. Target sequences were amplified with primers harboring the T7 promoter sequence. PCR products were purified using the Millipore Vacuum manifold system and checked for size and product specificity by agarose gel electrophoresis (top panel). In cases where more than one product was amplified, the product of the correct size was excised from the gel and purified. These products were then checked again by agarose gel electrophoresis (top right panel). To confirm the integrity of dsRNA synthesized in vitro from the PCR product templates, we ran 1 µg dsRNA on a denaturing formaldehyde gel (lower panel). Predominant bands are consistent with the predicted size for denatured RNA. We suspect the minor slower-migrating bands are non-denatured dsRNAs (A). By using RTPCR in BG2-Nhtt(62Q)EGFP cells treated with candidate groups 1–3 dsRNAs, we confirmed that in each case, the target gene was reduced upon dsRNA treatment. All results were from the same experiment except ATPsyn-b (boxed), which was from a different experiment (B).
(1.59 MB TIF)

Figure S5 Confocal microscopy of RNAi-treated cells. BG2-Nhtt(62Q)EGFP cells treated with candidate groups 1–3 aggregation-suppressing (A) and aggregation-enhancing (B) dsRNAs. To demonstrate the increase in diffuse-expressing cells among aggregation-suppressors, the EGFP gain was increased to 400 compared with a gain setting of 300 for imaging the aggregation-enhancing dsRNAs.
(6.00 MB TIF)

Figure S6 Confocal projection images of 3rd instar larval eye imaginal discs. Wandering 3rd instar larval eye discs were dissected in PBS, fixed in 4% PFA, stained with Hoechst and mounted onto a microscope slide in 80% glycerol for imaging using a Leica SP2 confocal microscope. Flies expressing Nhtt(18Q)EGFP together with Nhtt(18Q)EGFPNLS show localization of the protein in the nucleus (white arrow heads) and in the cytoplasm (white arrows) (left panels). Flies expressing mutant Nhtt(152Q)EGFP together with Nhtt(48Q)EGFPNLS show the presence of EGFP-positive nuclear (arrow heads) and cytoplasmic (white arrows) inclusions in larval eye imaginal discs (right panels). Images represent projection stacks of 5 µm z sections.
(2.47 MB TIF)

Figure S7 Correlation between inclusion number/load and cell viability. Based on the data shown in Table 1, the values of % viability were plotted against those of fold change in the number of inclusions or inclusion load for all candidate genes. A weak positive correlation was observed between cell viability and both number of inclusions (r = 0.436, P = 0.048) and inclusion load (r = 0.444, P = 0.044).
(2.46 MB TIF)

Figure S8 Functional grouping of mammalian orthologues of the candidate genes. The mammalian orthologues of the candidate genes (Table 1) identified by RNAi screening were categorized according to their known or predicted functions manually retrieved from the public databases such as PUBMED, Entrez Gene, and HomoloGene. Mammalian orthologues of enhancers and suppressors dsRNAs in the fly are shown by red- and blue-colored circles, respectively.
(3.79 MB TIF)

Data S1 Excel file: Full List Functional Assign Worksheet: 2SDs full list All candidates with z scores of greater than 2 or less than −2 are shown in this worksheet, with data for Gene Ontology categorization and manual functional grouping. The effect on mutant Htt aggregation is abbreviated as either enhancing aggregation (en) or suppressing aggregation (su). The predicted number of 19 nt OTEs and the mean % viability values are shown. Given the continual updating of public databases, current information may differ from information obtained at the time this spreadsheet was made. Worksheet: en 1.5 to 2 Weaker enhancer dsRNAs with z scores between 1.5 and 2 are shown with all the data entries as above.
(0.51 MB XLS)
