# Supplementary material for: RNAi Screening in Drosophila Cells Identifies New Modifiers of Mutant Huntingtin Aggregation
Source: PLoS One. 2009 Sep 30;4(9):e7275. doi: 10.1371/journal.pone.0007275 (PMC2748703; doi:10.1371/journal.pone.0007275)
Supplement: Text S1 — Supporting Materials and Methods (0.03 MB DOC) [file pone.0007275.s001.doc]

**Text S1: Supporting Materials and Methods**

**Cloning of *Drosophila* expression constructs**

N-terminal Htt exon 1 with 18Q, 62Q or 152Q as fusions with EGFP and containing a C terminal NLS and MYC tag, were excised from pIND mammalian expression constructs (Wang et al. 1999) and directionally cloned into the EcoRI/XbaI sites of pUAST. N-terminal Htt exon 1 with 18Q, 62Q or 152Q as fusions with EGFP alone were excised from the same mammalian expression constructs by EcoRI/BamHI digestion. The EGFP cDNA was amplified from the same mammalian construct using a primer than spanned the BamHI site between the Nhtt sequence and the EGFP coding region. Primer NhttBamHI-F 5’- CTGCACCGACC*GGATCC*ACCG – 3’ and primer 3’NhttEGFPstop 5’ – GGACGAGCTGTACAAGAAA**TAG***TCTAGAGGTACC*GGC – 3’ . BamHI, XbaI and KpnI restriction sites are italicized. The EGFP PCR product was cloned into the blunt EcoRV site of pBluescript SK(-) and subsequently excised with BamHI/XbaI or BamHI/KpnI. Full-length NhttEGFP clones were constructed in pUAST and pRMHa3 by 3 product ligation between the vector backbone, Nhtt 18, 62 or 152Q (EcoRI/BamHI), and EGFP (BamHI/XbaI or BamHI/KpnI for pUAST or pRMHa3 respectively).

**dsRNA library synthesis**

*In vitro* transcription reactions were set up in 96 well U-bottom plates (Cellstar) using Ambion T7 megascript kits to simultaneously synthesize sense and antisense RNA strands in one reaction. Following overnight synthesis, dsRNAs were purified using the Millipore manifold vacuum purification system® as per the manufacturer’s directions. The resulting dsRNAs, in 50μl TE, were stored at -70°C. The yields of randomly selected products were estimated based on spectrophotometer readings. We checked the size and integrity of a sample of dsRNA products by denaturing formaldehyde gel electrophoresis. Random samples were selected for dsRNA quantification by spectrophotometry to calculate the average product yield. The average product size was around 560 bp and the integrity was good using the protocol described. For screening, 2μl each dsRNA was seeded into duplicate 96-well plates and stored at -70°C until needed.

**Large-scale RNAi Screening**

10^4^ uninduced BG2-Nhtt(62Q)EGFP clone 1 cells in 100 μl complete Schneider’s *Drosophila* medium (Invitrogen) supplemented with 10μg/μl insulin (Sigma) and 10% FBS were seeded into 96 well plates containing dsRNA. Cells were agitated by hand for 30 seconds and incubated for 48 hours at 25°C. The average concentration of dsRNA was estimated to be around 109 nM. Although a lower concentration may be sufficient for knockdown of many genes, we wanted to ensure sufficient knockdown of most transcripts targeted, including those where the yield may have been lower than the average. Furthermore, we opted for shorter treatment with higher dsRNA concentration, in contrast to others who carry out RNAi screening. Expression of the Nhtt(62Q)EGFP was induced by the addition of 50 μl of 1.5 mM CuSO4 (final concentration 0.5mM). 18 hours later, cells were fixed by adding an equal volume (150 μl) of 8% PFA/0.36% Triton X-100 and nuclei subsequently stained with 5μg/ml Hoechst dye (Amersham Biosciences) diluted in PBS. Hoechst was removed after 10-20 minutes and the cells kept in PBS. The plate was analyzed by ArrayScan® and data for aggregate number, aggregate size and total cell number (based on the number of Hoechst-stained nuclei) was obtained. For tertiary screening and western blot analysis, cells were fixed and stained 16 hours post-induction.

**Statistical analysis and Batch Download GO information**

Each dsRNA screened was given a z score, based on raw data collected by the ArrayScan®. Each dsRNA was screened twice, with each experiment being carried independently. The z score was calculated by subtracting the plate mean from the dsRNA value for each measurement and dividing by the plate standard deviation (SD). ‘Hits’ were identified as those candidates with average z scores that were -2 or +2 standard deviations (SDs) away from the mean for at least one of the two data calculations (inclusion #/cell and/or inclusion load/cell). All data was entered into an excel spreadsheet. The CG ID number for each positive dsRNA was manually extracted from a spreadsheet provided by Open Biosystems and was used to search FlyBase Consortium. Using the batch download or batch search options, GO terms for Biological Process and Molecular function were extracted from FlyBase and FLIGHT database. Functional groups were manually assigned based on this information. For fly brain aggregation experiments, significance was determined using the Student’s t test comparing each candidate to the control.
