# Supplementary figures and images for: RNAi Screening in Drosophila Cells Identifies New Modifiers of Mutant Huntingtin Aggregation
Source: PLoS One. 2009 Sep 30;4(9):e7275. doi: 10.1371/journal.pone.0007275 (PMC2748703; doi:10.1371/journal.pone.0007275)

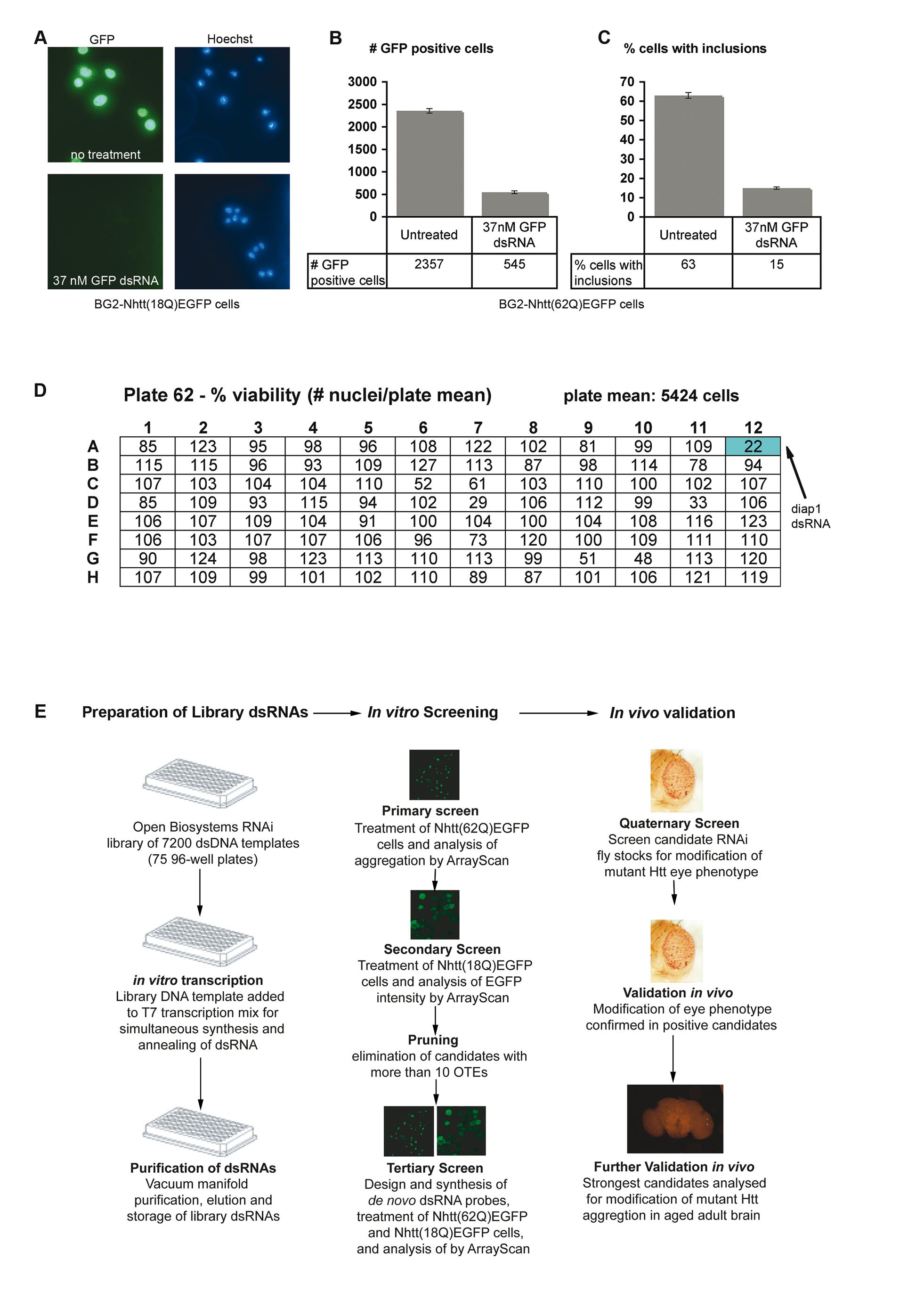

Supplement: Figure S1 — RNAi screening validation and overview. The efficacy of RNAi treatment in a Drosophila cell culture model of HD was tested using dsRNA against GFP. Cells expressing BG2-Nhtt(18Q)EGFP cells treated with 37 nM GFP dsRNA for 48 hours and visualized by fluorescence microscopy show ablation of GFP (A). BG2-Nhtt(62Q)EGFP cells were treated with GFP dsRNA and analyzed by ArrayScan®. Reduction of Nhtt(62Q)EGFP by GFP dsRNA reduced the number of EGFP-positive cells (B) and the number of EGFP-positive intracellular inclusions (C) detected by ArrayScan®. Screening in 96 well plate format was validated using a screen plate arrayed with random dsRNAs including dsRNA against diap1. Loss of diap1 results in widespread apoptosis as shown by the reduced viability in cells treated with diap1 dsRNA (D). An overview of our approach to screening for modifiers of mutant Htt aggregation, including several rounds of screening in vitro, followed by validation in vivo, is shown (E). (2.33 MB TIF) [file pone.0007275.s002.tif]

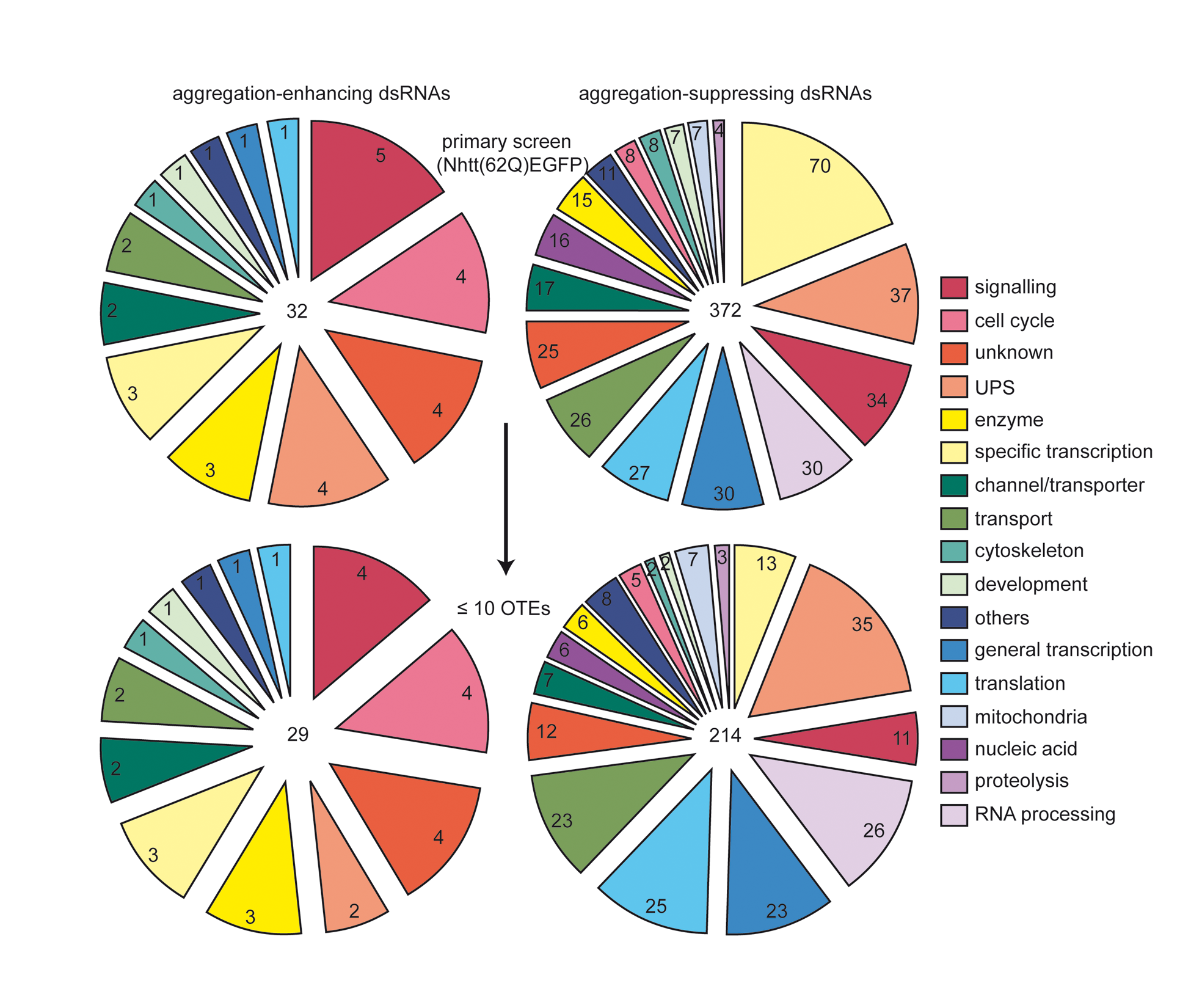

Supplement: Figure S2 — OTE pruning following primary screening. Top pie charts show the functional categorization of all candidates following primary screening in BG2-Nhtt(62)EGFP cells. These candidates were pruned to eliminated dsRNAs with more than 10 predicted OTEs. The Specific Transcription category, including many transcription factors (TFs) was the most drastically reduced following OTE pruning, consistent with the fact that many TFs have repetitive trinucleotide repeats that are sensitive to off-targeting. (1.81 MB TIF) [file pone.0007275.s003.tif]

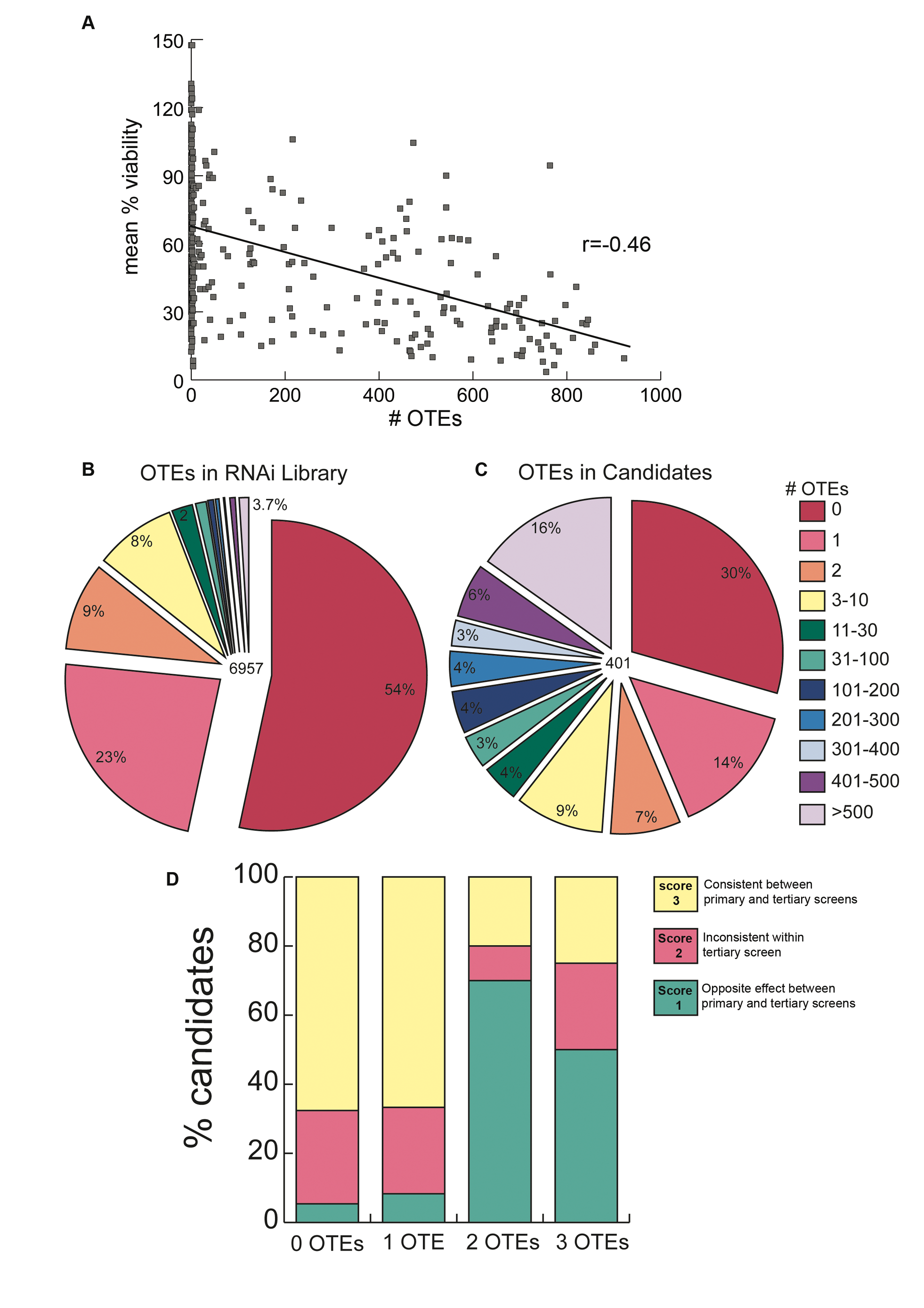

Supplement: Figure S3 — Off-target effects reduce cell viability and contribute to false positive candidates. The mean cell viability values for candidates following primary screening were plotted against the number of predicted 19 nt OTEs, demonstrating a significant negative correlation between cell viability and the number of potential OTEs (A). dsRNAs with multiple predicted OTEs are over-represented amongst our candidates following the primary screen. 54% of the dsRNA target sequences in the Open Biosystems library have no predicted 19 nt OTEs, with only 5.7% having more than 10 predicted OTEs (B). In contrast, 30% of our candidates lacked any predicted OTEs, with 44% having greater than 10 potential off-targets (C). The presence of off-target sequences causes inconsistencies in assay results. The percentage of candidates that modified Nhtt(62Q)EGFP consistently from the primary screen, using library dsRNAs, and the tertiary screen, using de novo designed dsRNAs are shown in yellow. The percentage of candidates producing an opposite effect is shown in green, while pink shows the percentage of candidates that gave inconsistent results within the tertiary screen duplicates using de novo dsRNAs with no 21 nt OTEs. The majority of candidates with no more than 1 OTE consistently modified mutant Htt aggregation in vitro. Increasing OTEs increased the likelihood of inconsistent results (D). (2.19 MB TIF) [file pone.0007275.s004.tif]

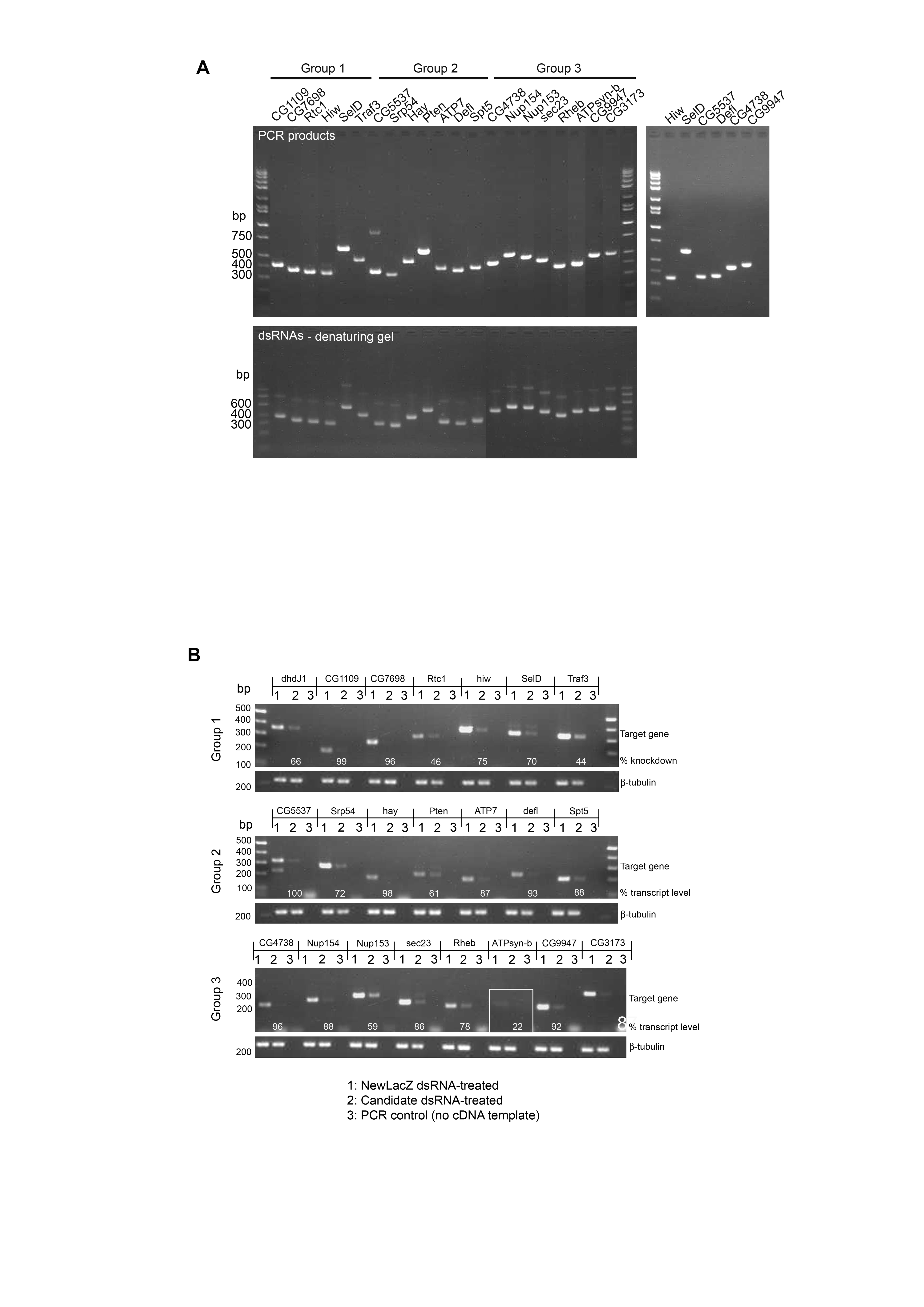

Supplement: Figure S4 — de novo RNAi probes. Target sequences were amplified with primers harboring the T7 promoter sequence. PCR products were purified using the Millipore Vacuum manifold system and checked for size and product specificity by agarose gel electrophoresis (top panel). In cases where more than one product was amplified, the product of the correct size was excised from the gel and purified. These products were then checked again by agarose gel electrophoresis (top right panel). To confirm the integrity of dsRNA synthesized in vitro from the PCR product templates, we ran 1 µg dsRNA on a denaturing formaldehyde gel (lower panel). Predominant bands are consistent with the predicted size for denatured RNA. We suspect the minor slower-migrating bands are non-denatured dsRNAs (A). By using RTPCR in BG2-Nhtt(62Q)EGFP cells treated with candidate groups 1–3 dsRNAs, we confirmed that in each case, the target gene was reduced upon dsRNA treatment. All results were from the same experiment except ATPsyn-b (boxed), which was from a different experiment (B). (1.59 MB TIF) [file pone.0007275.s005.tif]

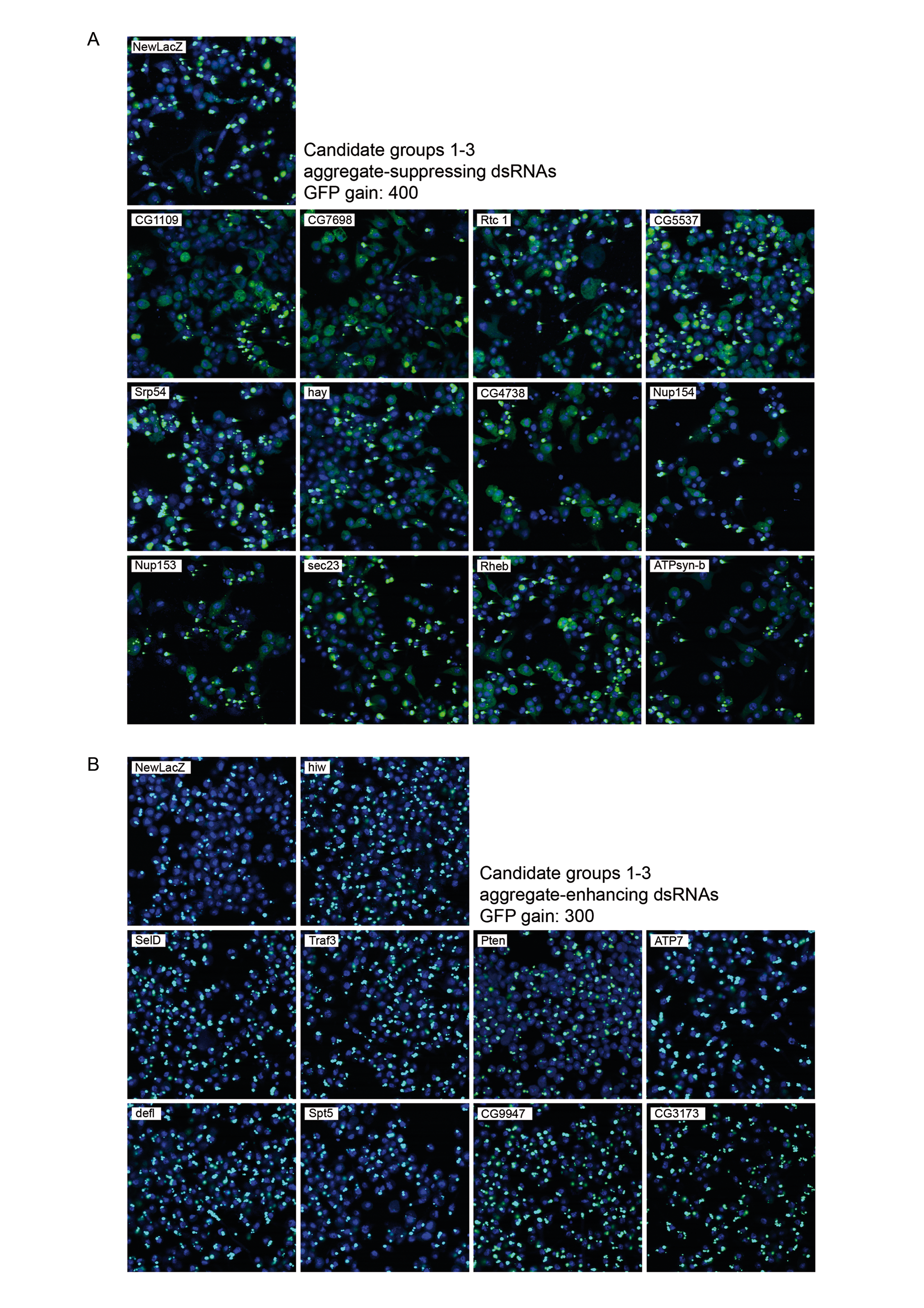

Supplement: Figure S5 — Confocal microscopy of RNAi-treated cells. BG2-Nhtt(62Q)EGFP cells treated with candidate groups 1–3 aggregation-suppressing (A) and aggregation-enhancing (B) dsRNAs. To demonstrate the increase in diffuse-expressing cells among aggregation-suppressors, the EGFP gain was increased to 400 compared with a gain setting of 300 for imaging the aggregation-enhancing dsRNAs. (6.00 MB TIF) [file pone.0007275.s006.tif]

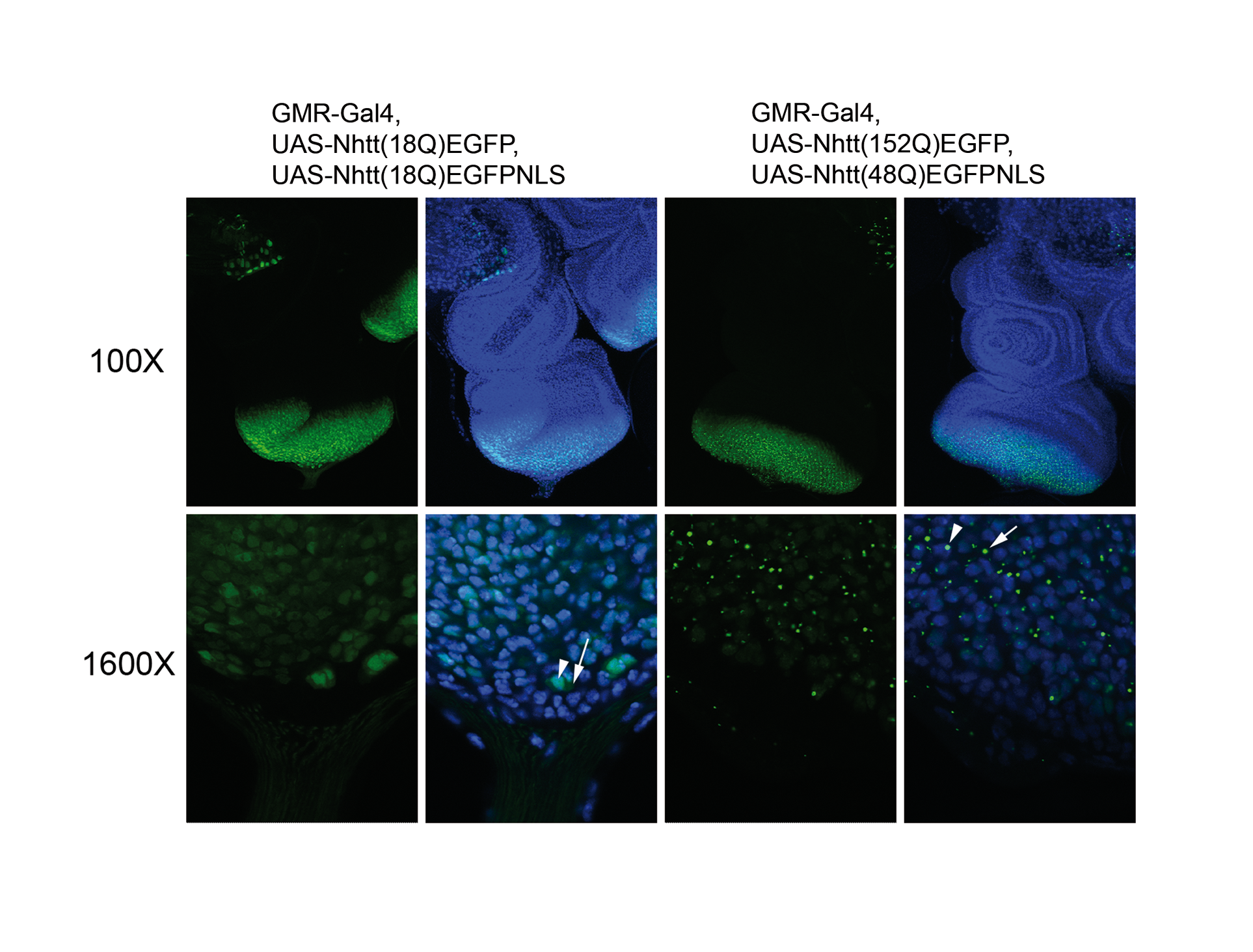

Supplement: Figure S6 — Confocal projection images of 3rd instar larval eye imaginal discs. Wandering 3rd instar larval eye discs were dissected in PBS, fixed in 4% PFA, stained with Hoechst and mounted onto a microscope slide in 80% glycerol for imaging using a Leica SP2 confocal microscope. Flies expressing Nhtt(18Q)EGFP together with Nhtt(18Q)EGFPNLS show localization of the protein in the nucleus (white arrow heads) and in the cytoplasm (white arrows) (left panels). Flies expressing mutant Nhtt(152Q)EGFP together with Nhtt(48Q)EGFPNLS show the presence of EGFP-positive nuclear (arrow heads) and cytoplasmic (white arrows) inclusions in larval eye imaginal discs (right panels). Images represent projection stacks of 5 µm z sections. (2.47 MB TIF) [file pone.0007275.s007.tif]

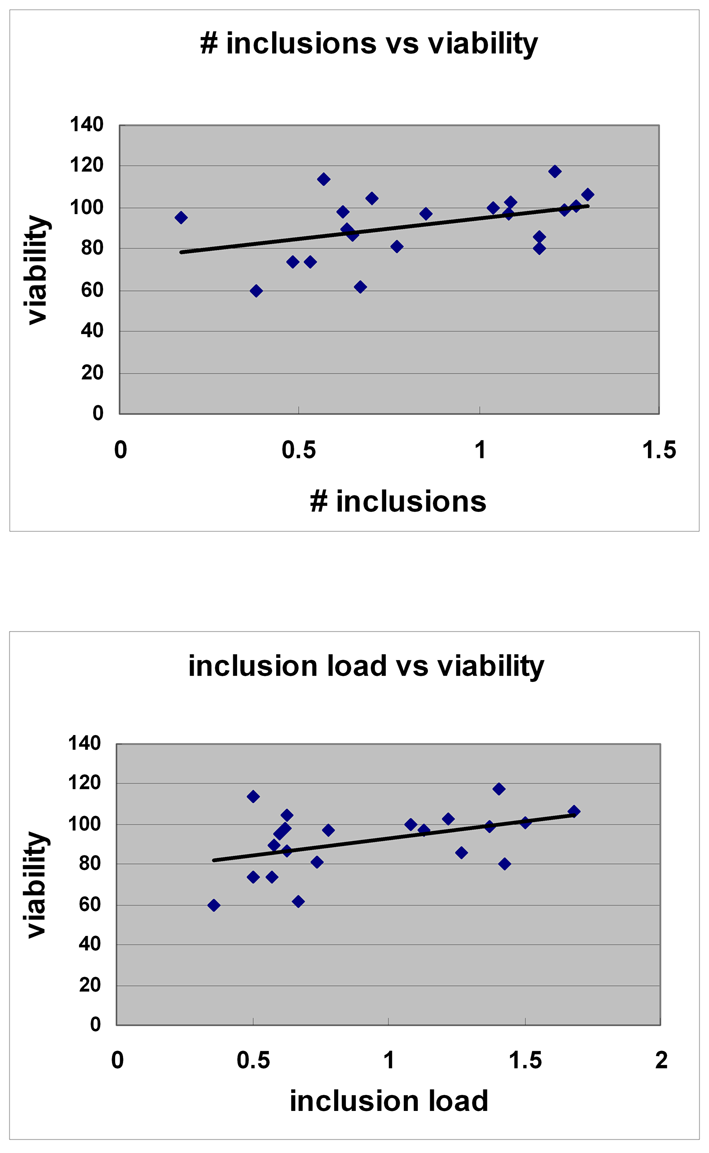

Supplement: Figure S7 — Correlation between inclusion number/load and cell viability. Based on the data shown in Table 1, the values of % viability were plotted against those of fold change in the number of inclusions or inclusion load for all candidate genes. A weak positive correlation was observed between cell viability and both number of inclusions (r = 0.436, P = 0.048) and inclusion load (r = 0.444, P = 0.044). (2.46 MB TIF) [file pone.0007275.s008.tif]

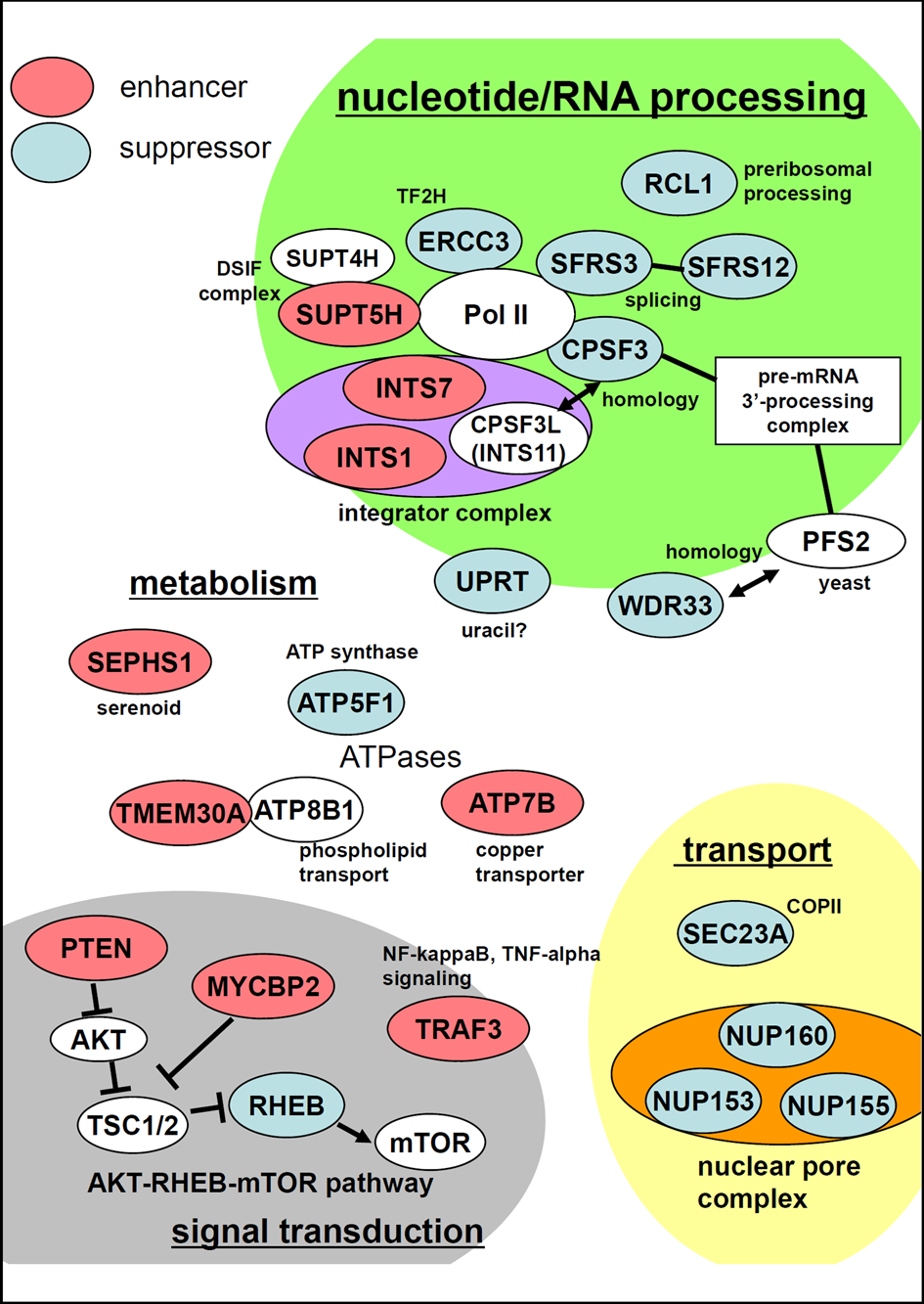

Supplement: Figure S8 — Functional grouping of mammalian orthologues of the candidate genes. The mammalian orthologues of the candidate genes (Table 1) identified by RNAi screening were categorized according to their known or predicted functions manually retrieved from the public databases such as PUBMED, Entrez Gene, and HomoloGene. Mammalian orthologues of enhancers and suppressors dsRNAs in the fly are shown by red- and blue-colored circles, respectively. (3.79 MB TIF) [file pone.0007275.s009.tif]
